# QoS-Aware Error Recovery in Wireless Body Sensor Networks Using Adaptive Network Coding

**DOI:** 10.3390/s150100440

**Published:** 2014-12-29

**Authors:** Mohammad Abdur Razzaque, Saeideh S. Javadi, Yahaya Coulibaly, Muta Tah Hira

**Affiliations:** 1 School of Computer Science and Statistics, Trinity College, Dublin 2, Ireland; 2 Faculty of Computing, Universiti Teknologi Malaysia, Skudai, 81310 Johor Bahru, Malaysia; E-Mails: javadi.saeideh@gmail.com (S.S.J.); cyahaya@gmail.com (Y.C.); 3 Faculty of Bioscience and Biomedical Engineering, Universiti Teknologi Malaysia, Skudai, 81310 Johor Bahru, Malaysia; E-Mail: hiraz105@gmail.com

**Keywords:** wireless body sensor networks, QoS, network coding, adaptability, reliability, energy efficiency

## Abstract

Wireless body sensor networks (WBSNs) for healthcare and medical applications are real-time and life-critical infrastructures, which require a strict guarantee of quality of service (QoS), in terms of latency, error rate and reliability. Considering the criticality of healthcare and medical applications, WBSNs need to fulfill users/applications and the corresponding network's QoS requirements. For instance, for a real-time application to support on-time data delivery, a WBSN needs to guarantee a constrained delay at the network level. A network coding-based error recovery mechanism is an emerging mechanism that can be used in these systems to support QoS at very low energy, memory and hardware cost. However, in dynamic network environments and user requirements, the original non-adaptive version of network coding fails to support some of the network and user QoS requirements. This work explores the QoS requirements of WBSNs in both perspectives of QoS. Based on these requirements, this paper proposes an adaptive network coding-based, QoS-aware error recovery mechanism for WBSNs. It utilizes network-level and user-/application-level information to make it adaptive in both contexts. Thus, it provides improved QoS support adaptively in terms of reliability, energy efficiency and delay. Simulation results show the potential of the proposed mechanism in terms of adaptability, reliability, real-time data delivery and network lifetime compared to its counterparts.

## 1. Introduction

Wireless body sensor networks (WBSNs) or body area networks (BANs) constitute an emerging and promising technology that will change people's healthcare experiences radically [1,2]. Unlike traditional healthcare systems, WBSNs will release patients from long hospital stays, thus reducing medical labor and infrastructural costs. In general, WBSNs will be cost effective, and they can continuously monitor physiological signals of patients, which will be very helpful, especially for the aging population [3]. Moreover, the use of WBSNs may enable ubiquitous healthcare and could lead to proactive and even remote diagnosis of diseases in an early stage. These systems provide uninterrupted health monitoring services, allowing patients to perform everyday activities, which lead to the enhancement of the quality of life [4]. However, wearable health monitoring technology is still young, and some challenging issues, such as quality of service (QoS), security and privacy, as well as social issues need to be resolved before this technology can be used widely. QoS is one of the main concerns for this technology. Generally, in network systems, such as WBSNs, QoS is viewed from two perspectives: the network and user/applications [5]. In the user/application perspective, QoS refers to an assurance of a set of requirements/services that are expected of the system by the users or applications. From the network perspective, QoS refers to a set of service qualities that the network offers to a user or application in terms of network QoS parameters, such as delay, reliability, energy efficiency, etc., during data delivery. As medical wearable systems deal with real-time and life-critical applications, they require a strict guarantee of QoS in both perspectives. To support QoS in WBSNs, the QoS requirements of these systems in user and network perspectives should be identified and addressed accordingly [6–10].

Addressing QoS in WBSNs is a difficult task. Some studies identified only reliability, real-time data delivery and network lifetime as QoS requirements of any wireless emergency system, such as WBSNs [2,11]. However, the QoS requirements in WBSNs are complex, and these systems may involve additional requirements in both perspectives of QoS [6–10]. Moreover, along with the resource constraints, these systems suffer from environmental and users' contexts in most applications, which pose additional challenges to support QoS [8]. For example, in hospital environments, very low signal-to-noise ratio (SNR) values are expected. Moreover, in and on body dynamic path loss [12] and patient's mobility increase the probability of packet loss in these systems. All of these issues constrain the solution space for QoS support in WBSNs and need to be considered in designing a QoS mechanism and framework (mostly media access control or MAC protocol centered) for these systems [6,8,10]. Existing works are available in the literature [13–17] that deal with QoS support in WBSNs. In these works, one or more of the necessary QoS requirements in health monitoring systems are missing. For example, reliable data delivery is covered in the proposed framework by Zhou *et al.* [17], but this requirement is missing in the work by Otal *et al.* [15]. Energy-saving MAC by Otal *et al.* [15] addresses real-time data delivery, but the work in [17] does not address this QoS requirement. Moreover, these studies do not consider environmental issues in designing their frameworks, and they may not be applicable in providing QoS support for real WBSNs [6,8,10].

A set of mechanisms (e.g., error recovery, clustering, power control) is available that can deal with QoS issues in WBSNs. Error recovery mechanisms are a very strong candidate to support QoS in WBSNs, as they can avoid retransmissions and improve QoS in terms of reliability, energy efficiency and real-time data delivery [11,18–20]. WBSNs need QoS mechanisms that can act as a function of (adaptive) network channel conditions [10,18]. However, traditional error recovery mechanisms, such as ARQ (automatic repeat-request) and FEC (forward error correction) are very hard to make adaptive [21]. Moreover, due to resource limitations in WBSNs, the use of complex and highly resource-hungry error recovery schemes, like ARQ and FEC, is undesirable in these systems [19]. Network coding (NC) is a new paradigm of protocol design in which intermediate nodes actively mix input packets to produce output packets. The size of the combined packet is the same as the size of an incoming packet. Moreover, XoR-based (⊕) NC mechanisms are simple and have no transmission overhead [22]. Thus, these can be used as an error recovery mechanism in wireless networks, including WBSNs [13,23–27], to improve network QoS parameters, such as reliability, energy efficiency and real-time data delivery, at very low energy, memory and hardware costs [13,23,24,26,28].

Most existing works [23–28] on NC for WBSNs include cooperative communications along with NC. In WBSNs, opportunistic [25] cooperation is not suitable; hence, dedicated relay or cooperative nodes are necessary [13,23–27]. The decode and forward mechanism-based works [26,29] are vulnerable to security and privacy attacks [30,31]. Clustering may not be possible in many applications of WBSNs, and the hierarchical clustering-based NC works [24,27] will not be useful. On the other hand, considering the complexity of ARQ [21], NC-integrated ARQ schemes [23] are not suitable for WBSNs. The authors in [13] have presented an NC-based error recovery scheme for WBSNs, which is not adaptive to network channel conditions or environments of WBSNs and user requirements. Most importantly, the majority of the existing NC-based works in WBSNs are unaware of the QoS requirements of applications.

The main objective of this work is three-fold: (i) to find out the QoS requirements of WBSNs from user and network perspectives and to identify the metrics and parameters that quantify those QoS requirements; (ii) to propose an NC-based error recovery mechanism for WBSNs that is adaptive to network channel conditions and users'/applications' requirements to support identified QoS requirements for WBSNs; and (iii) to compare the proposed mechanism with its counterparts in terms of adaptability, reliability, delay and energy efficiency. The proposed error recovery mechanism will be based on an existing work [13] rather than starting from scratch. Unlike [13], the proposed mechanism will be adaptive to channel conditions and user's demands to support QoS in dynamic network environments and user requirements of WBSNs.

Section 2 provides a brief overview on the analysis of QoS requirements of WBSNs from user and network perspectives. This section also identifies the metrics and parameters that quantify those QoS requirements. Section 3 briefly presents the related work. Section 4 presents the proposed NC-based error recovery mechanism for WBSNs that is adaptive to network channel conditions and users' requirements in supporting the necessary QoS identified in Section 2. The evaluation in Section 5 shows the potential of the proposed approach, and Section 6 concludes the work with some future directions.

## 2. Wireless Body Sensor Networks and QoS

### 2.1. Wireless Body Sensor Networks

**Figure 1.**
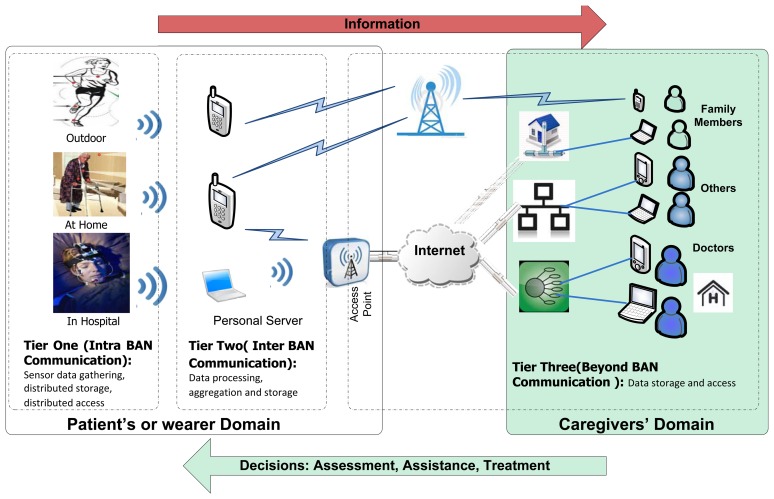
Structural design of wearable health monitoring system architecture.

**Figure 2.**
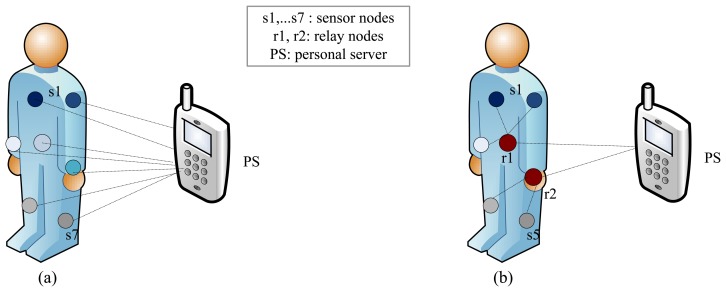
Wearable health monitoring system architecture: (**a**) Flat architecture of BAN, (**b**) Hierarchical architecture of BAN.

A WBSN consists of low-power, lightweight, small-sized and intelligent sensors that are placed in/on the human body. A general three-tier structural design for a WBSN is shown in Figure 1. The communication architecture between Tier 1 and the personal server (PS) can be flat or hierarchical, as illustrated in Figure 2a,b, respectively [2]. Studies [32,33] have shown that for QoS, a hierarchical architecture is preferable compared to the flat one, as the hierarchical architecture in WBSNs or BANs shows better network performance in terms of delay, packet delivery ratio (PDR), energy consumption and network lifetime.

### 2.2. QoS in Wireless Body Sensor Networks

As mentioned earlier, QoS requirements in WBSNs can be viewed from a user and network perspective. This section discusses the QoS requirements from both perspectives. In order to identify the QoS requirements from the network perspective, we rely on the literature. To gather information on the user point of view, we have selected a case study in the University Technology Malaysia's Health Centre [34] and Hospital Sultanah Aminah, Johor Bahru, Malaysia [35]. In the following, we briefly explain both views of QoS.

#### 2.2.1. QoS Requirements from the Network Perspective

In identifying QoS requirements, we rely on the type of applications, especially their data delivery model from sensors to the base station, which can be: continuous, query driven, event driven and hybrid [18,36,37]. Each of these data delivery models has its own QoS requirements. Table 1 summarizes the key QoS requirements for each data delivery model and its associated QoS metrics. As shown in the table, all of the data delivery models, apart from the continuous one, need all four key requirements, including real-time delivery, reliable delivery, energy efficiency and adaptability to the network's channel conditions. Typically, the continuous data delivery model can tolerate some delay and packet losses. If the communication channel suffers from a high rate of packet loss, QoS frameworks or mechanisms are required to take special care in transmitting critical data (e.g., events like a heart attack). This is to make sure that critical data are received at the destination correctly and on time [18,36,37].

#### 2.2.2. QoS Requirements from the Users' Perspective

For the user or application perspective of QoS, we have considered a case study that includes three medical application areas, including asthma and allergy, cardiovascular and diabetes. Medical specialists and nurses were selected from the three respective departments of the selected hospital and the health center. Based on their responses, we have summarized the sensor level priorities of the four chosen applications in Table 2. These sensor priorities are based on application or user's (patient's) demand. Along with the literature [2,6,11], respondents in the case study agreed that reliable and real-time data delivery are the two major QoS requirements in WBSNs or BANs. Table 3 summarizes the key QoS requirements in WBSNs and their related performance metrics.

#### 2.2.3. Mechanisms for QoS in WBSNs

Any method that improves the system performance in terms of QoS can be considered as a QoS mechanism, and a number of such mechanisms are available [10]. Table 4 summarizes these mechanisms along with their supported QoS. Adaptability includes user level adaptability, as well as the network's level. Existing works in WBSNs may include one or more of these mechanisms. For instance, the authors in [11,18,19] have considered collision management, error control and service differentiation. The dynamic nature of wireless links or channels of WBSNs or BANs is a challenging issue in providing QoS. Hence, from the QoS perspective, MAC protocols are more important than others (e.g., routing protocols, transport protocols). It is clear from Table 4 and existing works [10,11,18–20] that error recovery is one of the strongest mechanisms that can support most of the QoS requirements listed in Table 3, including network lifetime and real-time data delivery by avoiding retransmissions. ARQ, FEC and NC are the three main error recovery mechanisms.

## 3. Related Work

Two sets of existing works are related to this work. The first set of works is on QoS frameworks or mechanisms and the second one on NC-based works for WBSNs. In [14], the authors have improved the reliability of WBSNs in the case of node or links failures. They used clustering to improve reliability, energy efficiency and data delivery time. Their work does not support service differentiation, and it is not adaptive to dynamic network conditions. The distributed queuing body area network (DQBAN) [15] uses a cross-layer fuzzy rule-based scheduling algorithm to improve energy efficiency in WBSNs. It uses a QoS scheduler along with fuzzy logic rules implemented in each body sensor to avoid collisions. The deployed fuzzy logic approach in each body sensors helps with making a decision based on the SNR of the channel, residual battery power, *etc.* However, the integration of a fuzzy logic system in each body sensor makes system implementation complex and resource hungry, which makes this framework unsuitable for WBSNs [2]. Moreover, this work did not include sensor-level priority to support users' or applications' preferences. BodyQoS [17] provides QoS in WBSNs by applying service differentiation, radio-agnostic QoS and adaptive bandwidth scheduling. BodyQoS consists of three components: admission control, QoS scheduler and virtual MAC (VMAC). It estimates the effective bandwidth of a channel and if the channel is affected by noise and a high packet loss rate (PLR), then high priority sensors receive more resources to retransmit their data until they are transmitted correctly. However, too many retransmissions could make it unsuitable for delay-bounded applications. Retransmissions also may lead to buffer overflow in body sensor nodes and waste channel resources. Thus, the work may fail to provide reliable and real-time data delivery. In [38], the authors presented the urgency-based MAC (U-MAC) protocol. Body sensors that report urgent health information are given higher priority. High priority sensors get more retransmission slots by cutting-off from low priority body sensors. Thus, U-MAC improves the delivery time of high priority sensors. Like DQBAN, [38] does not consider dynamic channel conditions in the case of high packet loss probability, hence reliability. Moreover, the service differentiation considered is not adaptive to applications or users. Similarly, in PMAC (priority MAC) [39], protocol data channels are separated from control channels, and the priority is given to the life-critical traffic (emergency traffic). It also uses sleep mode for energy efficiency. Reliability in the case of dynamic and lossy channel conditions is missing. The authors in [40] considered traffic priority and load-adaptive MAC (PLA-MAC), which provides QoS to the packets according to their priority. The packets with higher priority get better services than the packets with lower priority. Even though it considers packet-level priority and reliability, the network's channel condition-based adaptation is missing. In [41], the authors have proposed a hybrid polling medium access control (MAC) protocol with human energy harvesting abilities (HEH-BMAC) for WBSNs. HEH-BMAC supports sensor-level priorities (high and normal), energy-awareness and flexibility. Other QoS issues, especially reliability, and adaptiveness to network's condition are missing.

In [26,29], the authors exploit the decode and forward mechanism for cooperation, which can be a concern for real-time applications. Importantly, decoding of every single packet at relay nodes is vulnerable to security and privacy attacks [30,31]. Use of clustering may not be possible in all applications of WBSNs, hence the hierarchical clustering-based NC works [24,27]. Due to the complexity of ARQ [21], NC-integrated ARQ schemes [23,42] are not suitable for WBSNs. The NC-based MAC proposed in [42] may not be suitable for WBSNs, as it is designed for wireless sensor networks. Moreover, some of the NC-based works [13] in BANs or WBSNs are not adaptive to the network channel conditions or environments of WBSNs. Most existing NC-based works in WBSNs exploit either linear combinations [24,27] or the XoR [13,26] operation for coding. Security-wise XoR-based coding is better than linear combinations. In a recent work [43], the authors proposed a cloud-assisted RLNC-based (random linear network coding) MAC protocol (CLNC-MAC). It supports guaranteed packet delivery and collision-free relaying, but suffers due to complexity and delay. In summary, most existing NC-based error recovery or performance improvement mechanisms are not QoS-aware of healthcare applications. Existing works which are QoS-aware do not support QoS in both perspectives, and they could be complex (e.g., [43]). In healthcare applications, inclusion of QoS awareness within these mechanisms is highly necessary.

## 4. Overview of the Proposed Error Recovery Mechanism

The work in [14] is the non-NC based error recovery mechanism that covers the widest range of QoS requirements compared to others. However, it is not adaptive to the network's channel conditions. Moreover, [14] applies a clustering mechanism, and it is very difficult to make a clustering algorithm adaptive to the network's dynamic channel condition. On the other hand, NC-based error recovery mechanisms [13,13,23–27] have great potential to be efficiently adaptive to the network channel conditions. Considering the security issue, this work will consider XoR-based NC for WBSNs. However, XoR-based existing works [26,29] could suffer in security and privacy, too, as they need to decode all or most of the packets at relay nodes. Therefore, Marinkovic and Popovici's (MP's) XoR coding-based approach [13] is selected as the base work in this work. This work will extend this NC-based mechanism in order to make it adaptive to the network's channel conditions and user QoS requirements. Adaptiveness to network channel condition and users' requirements will help the mechanism to improve the performance in terms of reliability, energy efficiency and real-time delivery. As the proposed work is based on MP's work, in the following, first, we briefly present MP's mechanism (for details, please see [13]) and then present the proposed one.

### 4.1. Network (WBSN) Scenario

We have chosen the hierarchical architecture of WBSN, which consists of different biomedical sensor nodes, Personal Server(PS)/monitoring station (MS) and relay nodes in between sensor nodes and PS (e.g., Figure 2b) [44,45]. The network can be modeled as a connectivity graph using the following equation,

(1) G=(V,E)

where *V* is the union of *S*, *R* and *PS*, *S* is the set of all *N* biomedical sensor nodes *S* = {*s*_1_, *s*_2_,…, *s_N_*} and *E* is the set of all wireless links between any biomedical sensor node and a relay node, any two relay nodes and/or between relay node and base station or sink, *E* = {*e*_1_, *e*_2_, *e*_3_,.., *e_N_*}, while *R* is the set of *n* relay node, *R* = {*r*_1_, *r*_2_, *r*_3_, …, *r_n_*}. Similar to MP's work [13], this work applies an NC-based error recovery mechanism for the wireless links between relay nodes and PS/MS.

**Figure 3.**
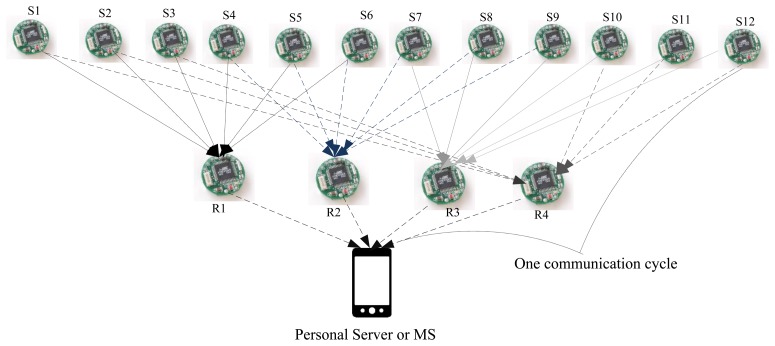
The network scenario used.

All of the medical sensor nodes in the considered WBSN use fixed and limited transmission power while communicating with relay nodes. Furthermore, the relay nodes use fixed and limited transmission power (higher than medical sensor nodes) during their communications with biomedical sensor nodes and PS/MS. In the considered WBSN, the medical sensor nodes act as source nodes and the relay nodes act as forwarding nodes. Typically, the placement of sensors in or on a human body is deterministic, hence the physical topology of the network. Thus, the WBSN, as in Figure 3, seems static, but in reality, it could be dynamic, due to its application environment (e.g., hospital, home) and in-body and on-body dynamic path loss [12]. The path loss modeling is out of the scope this paper, and we have considered an existing one similar to [12].

The appropriate number of relay nodes *n* depends on the application and the wearer's physical structure. Like MP's work, this work has considered that for a WBSN of 3*n* body sensors (S) and one monitoring station (MS or PS) with *n* >= 2, *n* relay nodes are sufficient. For the implementation of the proposed scheme, using simulation and analysis, if we considered a scenario that includes *n* = 4 or four relay nodes, 12 sensor nodes are sufficient (Figure 3) for the application.

### 4.2. Marinkovic and Popovici's Mechanism

**Figure 4.**
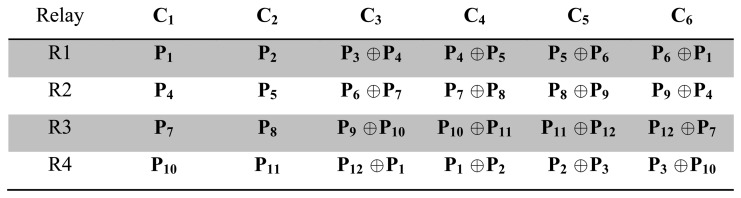
The packet coding schema used in Marinkovic and Popovici's (MP's) mechanism.

**Figure 5.**
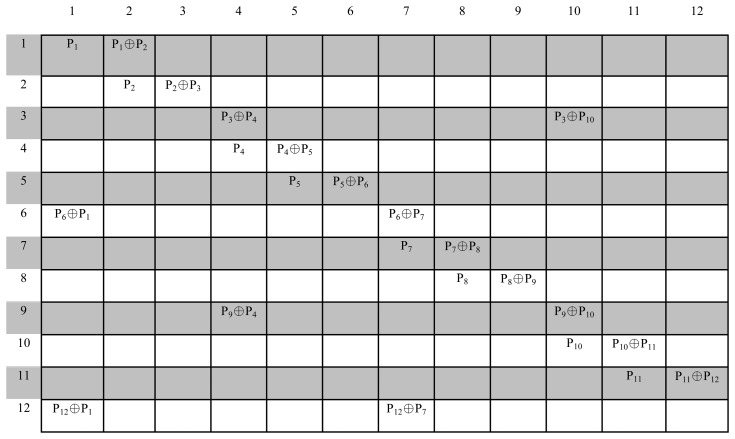
Received matrix at the destination.

In MP's NC-based error recovery mechanism, body sensors send their packets to the relay nodes, and the relay nodes XOR the received packets, then transmit them to the destination (PS). The network is designed in such way that each body sensor sends its data through two relay nodes and every relay node collects the data from six sensors (for the considered scenario). Sensor allocations are shown in Figure 3. The idea behind this approach is that every relay node sends two uncoded packets, and four packets coded using the XoR operation instead of sending six uncoded packets, as shown in Figure 4. With this approach, redundancies are available in the received packet, as seen (Figure 5) in the received matrix at the destination. The *N* × *N* received matrix in Figure 5 shows the available redundancy for every packet received from sensors, where *P_m_* represents the data packet from the sensor node *S_m_*, *m* = 1, 2, …, 12 and cell *P_m_*_×_*_m_* is the uncoded and *P_m_*_×_*_n_* the encoded packet, where *n* = 1, 2, …, 12. These redundancies ultimately help this approach to recover packets in the case of high PLR and improves the reliability. For instance (as shown in Figure 5), if packet *P*_1_ is lost, still, it can be recovered, as the destination might have received one or two encoded packets (*P*_12_ ⊕ *P*_1_ and *P*_1_ ⊕ *P*_2_), which contain *P*_1_.

### 4.3. The Proposed Error Recovery Mechanism

The strengths of NC and the weaknesses of the existing approaches, especially MP's [13] (summarized in Table 5), are the main motivation of the proposed error recovery mechanism. In order to improve MP's approach in terms of QoS performance, two QoS mechanisms are employed by this work: adaptive service differentiation and adaptive error recovery, which are briefly presented in the following subsections.

#### 4.3.1. Adaptive Service Differentiation

To address adaptiveness to users' or applications' requirements, an adaptive service differentiation mechanism has been included in the proposed approach. In this approach, two priority levels are defined: critical and non-critical (for simpli, we have considered two levels, but there can be more). Each sensor in a health monitoring system is labeled by one of these two priority levels. However, this priority assignment is not static, and users can reassign a priority level to sensors at any time depending on their needs. This functionality allows the users to identify the critical sensors for the system any time and get their responses according to their or the applications' demand. Thus, this QoS mechanism will be able to respond to users' or applications' queries in an adaptive fashion. The PS or MS executes the adaptive service differentiation algorithm and sends the priority information to the relay nodes, even to the sensors (not considered in this work) if necessary. In an application, the role of the relays is predefined, but can be changed in the next cycle if necessary. The change of role for the relays may require additional control overhead. As the relay nodes are comparatively powerful than sensor nodes, they can afford this overhead. Moreover, the role of relays in many healthcare applications may change very infrequently, which will minimize the overhead, and the impact on the overall performance will be little. The MS or PS assigns priority for every sensor depending on the application or the disease types and their senors' relative importance. The priority settings for each application can be preset (as shown in Table 2) or given by the doctors/nurses online. Here, we only consider critical and non-critical settings. Within each of this setting category (hierarchical), further leveling is possible.

#### 4.3.2. Adaptive Error Recovery Mechanism

One way to make an error recovery mechanism adaptive to the network channel conditions is to make the number of extra encoded packets adaptive based on the current packet error rate (PER) [10,18]. If the channel suffers from a high PER, the error control mechanism can provide the destination with extra encoded packets in order to increase the chance of packet recovery at the destination. This work relies on this method to make the proposed approach “adaptive to the network's channel conditions”. An error recovery mechanism integrated with service differentiation or sensor priority settings based on applications or user demand can improve the overall QoS support of the mechanism. Hence, the proposed error recovery mechanism along with PER also depends on the sensor's adaptive priority settings by the PS or MS.

**Figure 6.**
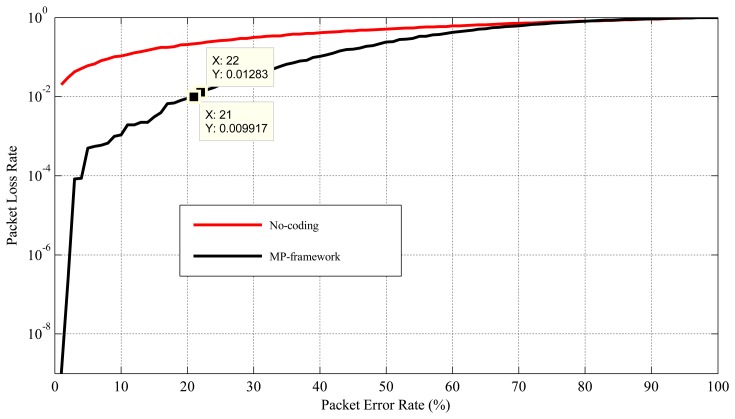
The MP mechanism's reliability performance.

For critical health monitoring applications, it is necessary to receive the critical data (high priority data) with a *PLR* < 1% [36,46]. However, MP's approach has failed to provide health monitoring applications with this requirement when the channel is suffering from a PER higher than 20% (as shown in Figure 6). This is because MP's error recovery mechanism is not adaptive to the network conditions, and it does not take necessary actions on transferring the high priority or critical data when the channel is suffering from a high rate of packet errors. In order to solve this problem, the proposed mechanism allows users or doctors to identify the critical sensors for the system or application. Every time a sensor sends its data to a relay node, the relay node checks its priority label (e.g., critical or non-critical). Then, considering the current PER from which the channel is suffering, it provides the destination with enough encoded packets for high priority data. This is to make sure that the destination receives the high priority data with a PLR under 1%. To add extra packets, the network is designed in such way that every relay node is responsible for making extra encoded packets for three sensors. For the considered network scenario, the allocations are: *R*1: *S*1, *S*2, *S*3, *R*2: *S*4, *S*5, *S*6, *R*3 : *S*7, *S*8, *S*9 and *R*4 : *S*10, *S*11, *S*12. Similar to MP's mechanism, in this work, every relay node collects data from six sensors (see Figure 3), but responsible for making “extra” encoded packets only for three sensors.

As shown in Figure 6, for PER between 1% to around 20–21%, MP's mechanism works fine for critical applications with *PLR* ≤ 1%. Therefore, no extra encoded packet is required to send to the destination for this range of PER. However, to support critical applications in the case of PER higher than 21%, few extra encoded packets are required. The calculation of the optimal number of extra encoded packets, based on the current PER and corresponding PLR, can be formulated as an optimization problem. Equation 2 represents the problem, where, *x* = the value of adaptive PER, *y* = the value of corresponding PLR and *P_nep_* = the number of extra encoded packets. The values of *PER* and *PLR* are expressed in the scale 0–1 instead of %.

(2) $$\begin{array}{ll} \underset{x}{\text{minimize}} & P_{\text{nep}}=f_{\text{nep}}(x,y)\\ \text{subject to} & 0.18\leq x\leq 1\\ & y\leq 0.01 \end{array}$$

To solve the above problem, a numerical method based on Algorithm 1 has been used. As shown in the algorithm, it iteratively finds the optimal number of extra encoded packets *P_nep_* required at the destination for PER between 18%–100%, both for critical and non-critical medical applications. For every value of PER higher than 18%, it first calculates the corresponding PLR. If the PLR is higher than the maximum allowed range (*PLR_cr_*_–_*_max_* or *PLR_ncr_*_–_*_max_*) of the application, then the destination is provided with one “extra” encoded packet for each high priority sensor datum. This continues until PLR or *y* satisfy the application requirement. From the numerical method, it is found that for the considered network scenario, extra encoded packets *P_nep_* for priority sensors vary from one to six, according to the PER. Equation 4 presents the minimum or optimum number of extra encoded packets required at the destination to achieve a PLR lower than 1% for high priority data. As every relay node (in the considered WBSN) receives the data only from six sensors, it cannot make more than five extra encoded packets. For example, R1 (Relay 1) receives the data from S1 (Sensor 1), S2, S3, S4, S5 and S6. This relay node is also responsible for making extra encoded packets for S1, S2 and S3. If S2 is a high priority sensor, R1 needs to make extra encoded packets for the data of S2. For this, the possible maximum number of combinations for R1 in providing the data of S2 is: *S*_1_ ⊕ *S*2, *S*3 ⊕ *S*2, *S*4 ⊕ *S*2, *S*5 ⊕ *S*2, *S*6 ⊕ *S*2 and *S*2 ⊕ *S*2. This is because XoR with itself (S2 ⊕ *S*2) provides zero. Hence, with the chosen network configuration 3, the proposed mechanism cannot provide the destination with more than five “extra” encoded packets and cannot provide the destination with an acceptable level of PLR for high priority data at PER higher than 52%. However, with a different network configuration, it will change.

(3) $$P_{\text{nep}}=\{\begin{array}{ll} 0 & \text{PER}<18\%\\ 1 & 18\%>\text{PER}\leq 30\%\\ 2 & 30\%>\text{PER}\leq 40\%\\ 3 & 40\%>\text{PER}\leq 45\%\\ 4 & 45\%>\text{PER}\leq 49\%\\ 5 & 49\%>\text{PER}\leq 52\%\\ 6 & 52\%>\text{PER} \end{array}$$

**Figure 7.**
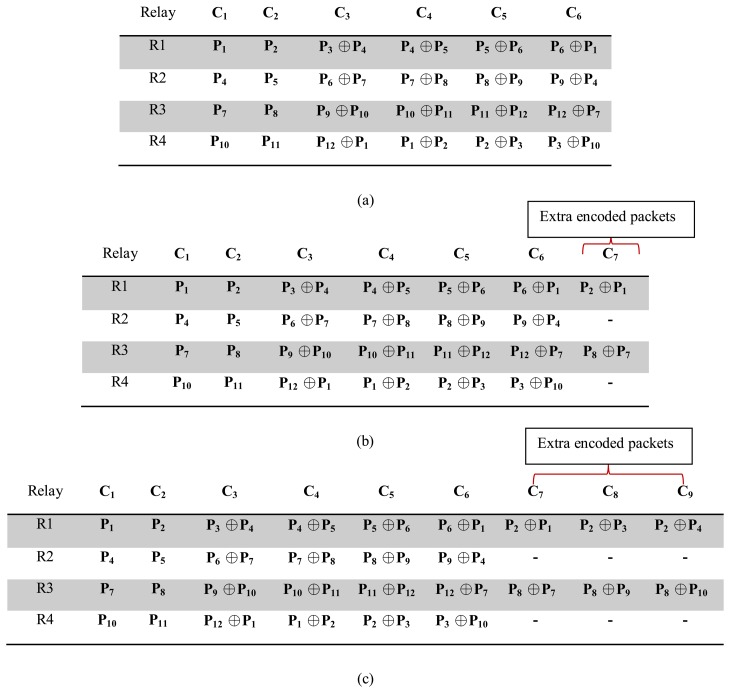
Packet coding schema for PER (in percentage) between (**a**) 0–18, (**b**) 18–30 and (**c**) 40–45 for S2 and S8 as critical sensors.

Let us explain a scenario to show how the proposed adaptive error recovery mechanism works. Consider that S8 and S2 are identified by users as critical sensors at the PS or MS, and this message is propagated to the relay nodes. After that, the relay nodes (the concerned relay nodes) find the number of required extra encoded packets based on the current PER from which the channel is suffering (using Algorithm 1). Finally, once they receive the data from the priority nodes S2 or S8, they add those extra encoded packets for these nodes' packets. Thus, at the destination, say PS or MS, more information is available to recover the data from S2 and S8. The recovery mechanism at the MS or PS is presented in Algorithm 2. Figure 7a–c presents the packet coding schema for PER between 0%–18%, 18%–30% and 40%–45% respectively, when S2 and S8 are labeled as critical sensors.


**Algorithm 1** : Network's channel condition and sensor priority-based adaptive encoding.
1:*x* : current PER value2:*y* : current PER's corresponding PLR3:*Ap_criticality_* : Criticality of the monitoring application type4:*P_nep_* : Optimal number of extra encoded packets5:*PLR_cr_*_–_*_max_* = .01, maximum allowed PLR for critical applications6:*PLR_ncr_*_–_*_max_* : maximum allowed PLR for non-critical applications7:**if**
*Ap_criticality_* = true **then**8: **while**
*y* ≥ *PLR_cr_*_–_*_max_*
**do**9:  *P_nep_* = *P_nep_* + 110:  read current PER11:  calculate corresponding PLR or *y*12: **end while**13:**else**14: **while**
*y* ≥ *PLR_ncr_*_–_*_max_*
**do**15:  *P_nep_* = *P_nep_* + 116:  read current PER17:  calculate corresponding PLR or *y*18: **end while**19:**end if**


#### 4.3.3. Recovery Process

**Figure 8.**
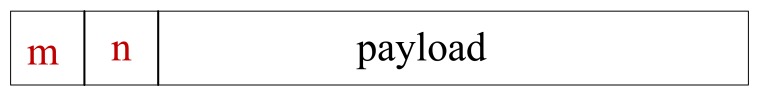
Packet format considered in the proposed mechanism.

Like the MP's mechanism, the proposed mechanism considers TDMA (time division multiple access) for channel access. When the sensor nodes send packets to the MS/PS using TDMA, the packets sent in the same TDMA frame are considered the same generation packets. Typically, in the relay nodes, the same generation's packets are encoded at the same time. Similarly, at the destination, the decoding process is done only with the packets of the same generation. For encoding, we have used the format shown in Figure 8, where the encoding vector {*m*, *n*} represents the sensor ID of the packets that are coded in the packet. For any packet with an encoding vector, where *m* ≠ *n*, the payload is: *P_mn_* = *P_m_* ⊕ *P_n_*; where *P_m_* and *P_n_* represent the packets received from the sensors *S_m_* and *S_n_*, and *m*, *n* = 1, 2, …, 12 (or maximum number of sensors). If *m* = *n*, the payload is *P_mm_* = *P_m_*, and this is the uncoded packet *P_m_* from sensor *S_m_*.

During each TDMA cycle, the MS/PS receives *P_i,j_* coded packets of the same generation and stores them in the memory. After that, they are buffered, and pointers (*A_i,j_*) to the first byte of each packet (*P_i_*,*_j_*) are written in the decoding matrix *D*(*n*, *n*) (shown in Equation 4).

**Figure 9.**
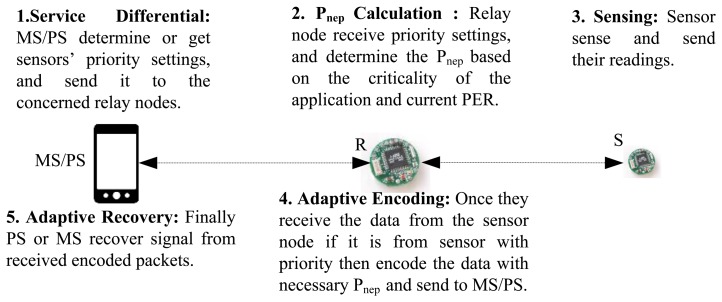
The working principle of the proposed error recovery mechanism.

At the end of every communication cycle, the recovery process (as shown in Figure 9) at MS/PS is done in two steps:

Step 1. As *P_m_* ⊕ *P_n_* = *P_n_* ⊕ *P_m_*, *D_nn_* (Equation 2) is updated in such way that: if *D*(*m*, *n*) ≠ 0, then *D*(*n*, *m*) = *D*(*m*, *n*), and both pointers point to the same packet *P_m,n_* = *P_n,m_*.
Step 2. As *P_m_* = *P_k_* ⊕ (*P_k_* ⊕ *P_m_*), packets are recovered using the following rule: if (*D*(*k*, *k*) ≠ 0 & *D*(*k*, *m*) ≠ 0), then *P_m_*,*_m_* = *P_k_*,*_k_* ⊕ *P_k_*,*_m_*; recovered packet *P_m,m_* is stored in buffer, and matrix *D*(*n*, *n*) is modified to contain the pointer to that packet *D*(*m*, *m*) = *A_m_*,*_m_*.

Step 2 is repeated until all of the packets are reconstructed. Once this is done, the matrix *D* is then emptied to make it ready for the next communication cycle. These steps are summarized as Algorithm 2.

(4) $$D(n,n)=\left(\begin{array}{ccccccc} A_{1,1} & A_{1,2} & 0 & 0 & A_{1,5} & \cdots & A_{1,n}\\ 0 & 0 & A_{2,3} & 0 & A_{2,5} & \cdots & 0\\ A_{3,1} & 0 & 0 & A_{3,4} & 0 & \cdots & 0\\ A_{4,1} & 0 & 0 & A_{4,4} & 0 & \cdots & 0\\ \vdots & & & \vdots & \vdots & & \\ A_{n,1} & 0 & 0 & A_{n,4} & 0 & \cdots & A_{n,n} \end{array}\right)$$

where:

*D*(*i*, *j*) = 0(*Null*): if *P_i_*,*_j_* was not received or does not exist;
*D*(*i*, *j*) ≠ 0, *i* = *j*: if *P_i_* was received and stored in memory;
*D*(*i*, *j*) ≠ 0, *i* ≠ *j*: if *P_i,j_*(= *P_i_* ⊕ *P_j_*) was reviewed and buffered.

**Algorithm 2** : Recovery process at Personal Server /monitoring station (MS).
1:*Step 1*:2:**for**
*i* = 1 to 12 **do**3: **for**
*j* = 1 to 12 **do**4:  **if**
*D*(*i*, *j*) ≠ 0 **then**5:   *D*(*j*, *i*) = *D*(*i*, *j*) {Both pointers point to the same packet}6:  **end if**7: **end for**8:**end for**9:*Step 2:*10:*V_repeat_* = 111:**while**
*V_repeat_* > 0 **do**12: **for**
*i* = 1 to 12 **do**13:  **if**
*D*(*i*, *i*) ≠ 0 **then**14:   **for**
*j* = 1 to 12 **do**15:    **if**
*D*(*i*, *j*) ≠ 0 & *D*(*j*, *j*) ≠ 0 **then**16:     *P_j_*,*_j_* = *P_i_*,*_i_* ⊕ *P_i_*,*_j_* {Recovered *P_j_* = *P_i_* ⊕ (*P_i_* ⊕ *P_j_*)}17:     *D*(*j*, *j*) = *Aj*, *j* {Packet *P_j_* is buffered and pointer in *D*(*n*, *n*)}18:     *V_repeat_* = 119:    **end if**20:   **end for**21:  **end if**22: **end for**23:**end while**


## 5. Performance Evaluation

### 5.1. Simulation Setup

As identified earlier, reliability, real-time data delivery and network lifetime are the three main QoS requirements for WBSN applications. To evaluate the improvement of QoS in WBSNs, particularly the improvement of reliability, real-time data delivery and network lifetime, we have used simulation. PLR, data delivery delay and energy efficiency or consumption are considered as the responsible metrics for the reliability, timeliness and network lifetime QoS requirements, respectively. Reliability is measured using the packet loss rate, or PLR, metric that is defined as the number of packets that cannot be recovered at the destination divided by the number of packets sent without any network coding. On the other hand, for the delay and the energy efficiency improvement, we have used the number of retransmissions needed for the mechanisms to receive and recover data. Usually, higher PER means more retransmissions and more delay and energy consumption at the relay nodes. Three mechanisms, the proposed one, the MP's one and no coding, were included in the comparative study. For the proposed scheme, three simulation results, one for the non-critical data, one for critical data and one for the overall or combined data, have been generated.

The considered WBSN simulation scenario consists of 12 sensors nodes and four relay nodes. All of the sensors continuously sense and send their readings to the destination or PS through the assigned relay nodes. As this work aims to employ NC for the links between the relay nodes and PS, it is assumed that the sensor readings are reaching the relay nodes without any error. Once, the sensor readings are available at the relay nodes, they check the priority of the sensors, encode them accordingly and send them to the PS. Sensor priority settings can be dynamically changed based on the requirements. To demonstrate the dynamic priority settings, MATLAB's pseudo-random generator was used. The key components of the implementations include the encoding process that resides in the relay nodes, the recovery process and the sensor priority allocation process, which reside in the PS/MS. To mimic the wireless channel's dynamic behavior, random PERs were generated for the links between the relays and PS using MATLAB's pseudo-random generator. Based on these PERs, PS calculates the corresponding PLR and *P_nep_* for each link and sends them to the relay nodes. If a packet from a sensor cannot be recovered, it is counted as lost, and relays in the next communication cycle send the next generation data from the sensor. As we have considered TDMA for channel access, each TDMA frame is considered as a cycle.

To get consistent results, the mechanisms were simulated 3000 times. During each simulation time, the PER was varied between 1%–100%, and the averages of 3000 simulations were selected for the results.

### 5.2. Results

**Figure 10.**
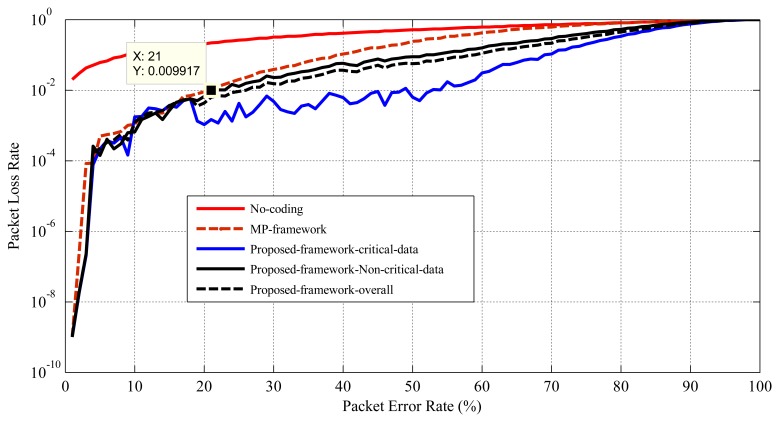
Performance comparison in terms of reliability.

Figure 10 presents the performance of this mechanism in terms of reliability. If the mechanism can maintain PLR below a certain threshold (1% for critical applications) in the case of variable PER, then it can maintain the communication reliability. As the proposed mechanism considers service differentiation, it is enabled to provide different PLRs for different groups of sensors (critical and non-critical). As we can see from Figure 10, the PLR of the critical data and the non-critical data for PER between 1%–18% is very similar to the MP's mechanism. This is because of the PER between 1%–18%; the proposed mechanism follows the same packet coding schema as the MP's mechanism (no extra encoded packets are generated for PER between 1% to 18%; see Figures 7a and 4). However, in the proposed mechanism for the PER higher than 18%, both the non-critical and the critical data experience better PLR compared to the MP's mechanism. As for the PER higher than 18%, the proposed mechanism provides the destination with some extra encoded packets in order to provide sufficient redundancy, so that the destination can receive the critical data with a PLR under 1% or 10^−2^ (an acceptable level). As shown in Figure 10, the proposed mechanism shows better PLR or reliability compared to the MP's mechanism and the no-coding scheme for PER up to 90%. However, it can provide health monitoring applications with an acceptable level of PLR for the critical data when the PER varies between 1% to about 52%. The reason for this behavior is explained in Section 4.3.2. Moreover, as we can see from Figure 10, for the critical data, the PLR fluctuates a bit with the PER, especially in the range of 20%–60%. One of the main reasons for this could be the strict and lower PER threshold ranges for critical applications and their corresponding extra encoded packet actions. For instance, around 28%–29% PER, the PLR increases, and it is detected and extra encoded packet action taken close to 30% PER, which decreases the PLR. This continues approximately up to 60% PER, and after that, the PLR increases and goes beyond the acceptable range. For 60% and above PER, no extra encoded packets are applied in the considered network scenario; hence, the PLR rises along with the PER.

A high level of reliability, especially in the case of high PER, can make the proposed mechanism a reliable one during the security attacks. Though PLR is a QoS issue, it has the potential to pose a serious security threat in WBSNs. In WBSNs environment [31], attackers can harm the system by simply presenting a low level of noise to the channel and causing a lot of packet losses. They even can block the whole system by causing infinite retransmissions. The interesting point in the reliability performance of this mechanism is that this mechanism generates extra encoded packets only in order to support the high priority data, but still, the non-critical data experience a better PLR. This is because of the XoR (⊕) coding schema that makes relation between different types of packets. For example, consider the scenario where S1 is labeled as the critical sensor and S6 is labeled as non-critical. Furthermore, consider that the channel is suffering from high values of PER and that both the S1 and S6 data are lost during the transmissions. However, as this mechanism provides the destination with few extra encoded packets related to S1's data, the destination can recover the S1 data (P1;. Moreover, Relay 1 always sends an XoRed packet that includes the data of S1 and S6 (*P*1⊕*P*6). Thus, when the personal server (destination) receives the *P*1 ⊕ *P*6, by having P1, it can recover the P6, too, as *P*6 = *P*1 ⊕ (*P*6 ⊕ *P*1).

**Figure 11.**
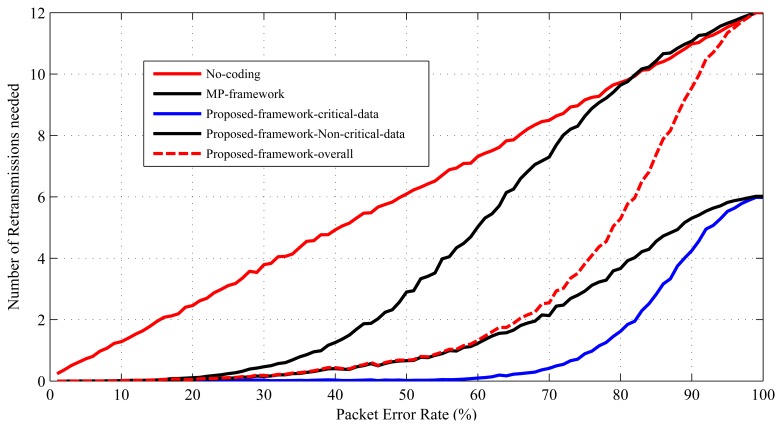
Performance comparison in terms of the number of retransmissions needed.

**Figure 12.**
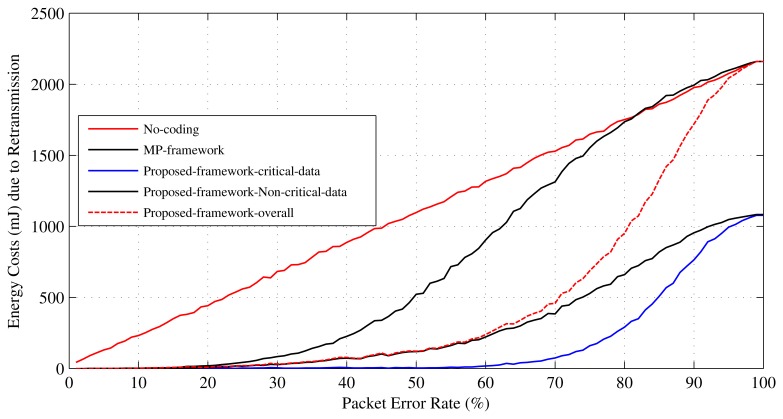
Energy consumed due to the retransmissions.

**Figure 13.**
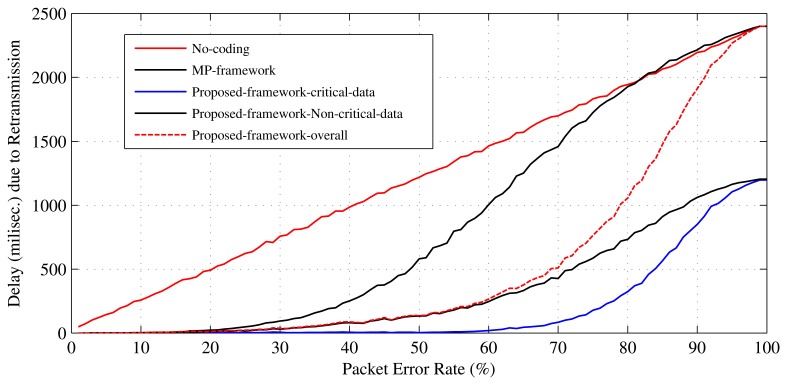
Data delivery delay due to the retransmissions.

The number of required retransmissions *N_rtx_* is a parameter that can be used to determine how much an error recovery mechanism improves QoS in terms of energy consumption and data delivery delay [10,11]. This work uses the *N_rtx_* parameter to evaluate the performance of the proposed error recovery mechanism in terms of energy efficiency and data delivery delay. Retransmissions cause extra energy, as well as delay in a WBSN. Therefore, the reduction in *N_rtx_* helps to make the proposed scheme energy efficient and to reduce delay in WBSNs. Figure 11 presents the performance analysis of the proposed mechanism in terms of the number of retransmissions needed by it. As presented in Figure 11, both the critical and the non-critical sensors in the proposed scheme require a smaller number of retransmissions in comparison to the MP's mechanism or without any error recovery method. This is because the probability of packet loss in this mechanism is lower than the others (see Figure 10). Due to the lower loss probability, the body sensors are required to retransmit their data less. If each retransmission consumes *T* units of time and *E* units of energy, it can be said that at 70% PER in MP's mechanism, the set of 12 sensors consumes about 8*T* units of time and 8*E* units of energy, and in the mechanism without error recovery, they consume 9*T* units of time and 9*E* units of energy. For the same settings, in the proposed mechanism, the sensors consume (overall) 4*T* units of time and 4*E* units of energy, where, individually, the set of non-critical sensors consume 3*T* units of time and 3*E* units of energy, and the set of critical sensors consume only one unit of time and one unit of energy. Figures 12 and 13 present the results for the energy efficiency and data delivery delay of the proposed mechanism considering the TelosB [47] as sensor nodes and the Imote2 [48] as relay nodes. We also considered that the distance between the relay nodes and personal server is around 20 meters. These figures clearly show the potential of the proposed mechanism in terms of energy efficiency and data delivery delay improvement. Trends in Figures 12 and 13 are very similar to Figure 11, as the energy costs and the delays are mainly contributed by the retransmissions.

## 6. Conclusions

Typically, healthcare applications are real time and life critical, which require a strict guarantee of quality of service (QoS), in terms of latency, error rate, reliability and security. Importantly, WBSNs need to provide QoS support from both (user and network) perspectives. In this context, this work has highlighted the key QoS requirements of WBSNs from the user and the network perspectives. It has also identified the metrics and the parameters that quantify these QoS requirements. These requirements include real-time data delivery, reliable data delivery, energy efficiency, adaptiveness to channel conditions and adaptiveness to user/application queries. It is also found that the majority of the existing QoS mechanisms in WBSNs do not support all of these QoS requirements. Furthermore, investigations have been carried out in this paper to show that the network coding-based MP's work fails to support adaptiveness to the channel conditions, and adaptiveness to the users'/applications' requirements, which are very important in critical medical applications (e.g., heart attack detection). Hence, to support these QoS requirements, a network coding-based error recovery mechanism integrated with a service differentiation scheme for WBSNs has been proposed in this work. This NC-based error recovery mechanism makes the encoding scheme at the relay nodes adaptive, which makes the framework adaptive to the network channel condition and the users' requirements. As the results show, along with the adaptiveness, the proposed mechanism has the potential to improve QoS support by performing fewer retransmissions. Moreover, the mechanism provides some security measures to protect patients' data. Reduced retransmissions ultimately help to reduce the delay and the energy consumption. Table 6 summarizes the key features of the proposed QoS mechanism along with a few existing ones.

In this work, as well as in the MP's work, NC has been used only for the links between relay nodes and PS/MS, not for the sensors to relay nodes. Therefore, the links, especially wireless links, between the sensors' and relay nodes' communication can be unreliable. Hence, the integration of NC at the sensor level is a recommended future research direction. In the evaluation, this work has relied on simulation rather than real sensor-level implementations. Real sensors and a sensor node-level study would be of merit.

## Figures and Tables

**Table 1.** Key QoS requirements and metrics for different delivery models.

**Delivery Model**	**Real-Time Delivery**	**Reliable Delivery**	**Energy Efficiency**	**Adaptability**
Event driven	✓	✓	✓	✓
Query driven	✓	✓	✓	✓
Continuous	-	-	✓	✓
Hybrid	✓	✓	✓	✓

**Table 2.** Importance level of physiological signals in some medical applications.

**Priority Level**	**Cardiovascular**	**Asthma and Allergy**	**Diabetic**	**Heart-Attack**
Level one	Blood pressure	Oxygen saturation (*S_p_O*_2_)	Blood glucose	ECG
Level two	ECG	*CO*_2_	Blood pressure	Blood pressure
Level three	Blood glucose	ECG	*S_p_O*_2_	*S_p_O*_2_
Level four	*S_p_O*_2_	Blood pressure	*CO*_2_	*CO*_2_
Level five	*CO*_2_	Blood glucose	ECG	Blood glucose
Level six	Gyroscope	Gyroscope	Temperature	Temperature
Level seven	Temperature	EEG	EEG	EEG
Level eight	EEG	Temperature	Gyroscope	Gyroscope

**Table 3.** QoS requirements for WBSNs from network and user perspectives.

**QoS Requirements**	**Network/User Perspective**	**Metric Involved**
Real-Time Data Delivery	Both	Delay
Reliable Data Delivery	Both	Packet loss rate (PLR)
Adaptive to user Queries	User/Application	Sensor priority
Network Lifetime	Network	Energy efficiency
Adaptive to Network Channel Conditions	Network	Packet error rate (PER)

**Table 4.** QoS mechanisms and the QoS requirements addressed by them.

	**QoS Requirements**			

**QoS Mechanism**	**Reliability**	**Real-Time Delivery**	**Energy Efficiency**	**Adaptability**
Collision Management	-	✓	✓	✓
Clustering	✓	✓	✓	✓
Data Compression	-	-	✓	✓
Error Recovery	✓	✓		✓
Power Control	✓	✓	✓	✓
Service Differentiation	-	-	-	✓

**Table 5.** The MP mechanism's performance analysis.

**Strengths**	**Weaknesses**
Improved Reliability	Not adaptive to network's channel condition
Improved delay in data delivery	Not adaptive to users' queries
Reduced energy consumption	Does not support service differentiation
Lightweight	Does not consider environmental context of WBSNs

**Table 6.** QoS requirements addressed by the proposed mechanism. U-MAC, urgency-based MAC; DQBAN, distributed queuing body area network.

**Supported QoS**					

**Mechanism**	**Real-Time Data Delivery**	**Reliability**	**Energy Efficiency**	**Adaptiveness to Channel Conditions**	**Adaptiveness to User Queries**
U-MAC [38]	for priority sensors	-	-	-	-
BodyQoS [17]	-	for priority sensors	-	yes	-
DQBAN [15]	-	-	yes	yes	-
Reliable WBAN [15]	yes	yes	yes	-	-
MP's [13]	yes	yes	yes	-	-
Proposed	yes	yes	yes	yes	yes
